# Antibacterial activity of silver nanoparticles of different particle size against *Vibrio Natriegens*

**DOI:** 10.1371/journal.pone.0222322

**Published:** 2019-09-13

**Authors:** Yaohua Dong, Hongling Zhu, Yuanyuan Shen, Wenting Zhang, Li Zhang

**Affiliations:** 1 College of Ocean Science and Engineering, Shanghai Maritime University, Shanghai, China; 2 School of Mechanical Engineering, Shanghai JiaoTong University, Shanghai, China; VIT University, INDIA

## Abstract

In this study, we describe the synthesis and characterization of silver nanoparticles (Ag-NPs) of different sizes and evaluated their antibacterial activity. Particles size and morphology were characterized by transmission electron microscopy. Evaluation of the bacteriostatic effects was performed by ultraviolet–visible spectrophotometry and comet assays. The smaller the particle size of Ag-NPs, the smaller the value of the minimum inhibitory concentration (MIC) and minimum bactericidal concentrations (MBC), indicating the greater the antibacterial activity. The antibacterial activity was determined by the generation of reactive oxygen species (ROS) by bacteria and by bacterial membrane damage. In this study, we determined ROS-induced damage of bacteria caused by Ag-NPs. In conclusion, our findings indicated that Ag-NPs were effective at different particle sizes and concentrations and that the smaller the particle size of Ag-NPs, the greater the antibacterial activity.

## Introduction

In the past decades, metallic nanoparticles (NPs) have received significant attention due to their unique physicochemical properties[1]. In this regard, silver (Ag) NPs are particularly outstanding because of their potential applications in health care[2], textile fibers[3], food packaging[4], and antibacterial fields[5]. Moreover, Ag-NPs are considered non-toxic or less harmful to mammalian tissues and environmentally friendly when used in relatively low concentrations at the same size and shape[6,7]. In recent years, extensive interrelated investigations have been performed to better utilize Ag-NPs for the inhibition of bacteria, however, there is an ongoing debate regarding the antibacterial mechanism of action of Ag-NPs. In previous studies, it has been indicated that the bactericidal properties of Ag-NPs primarily depend on the release of Ag+ ions[8,9] from NP surfaces, followed by the interaction of Ag+ ions with cellular targets[4]. Based on other studies, however, the release of Ag+ ions is not the critical factor for Ag-NP-induced toxicity[10], which may arise from direct physical processes caused by nano-objects, such as disruption of the cell membrane, penetration into the cytoplasm of bacteria[11], DNA replication[12], and ribosomal damage. Furthermore, several studies have reported that the antibacterial activity of Ag-NPs cannot solely be attributed to one aspect but rather to the comprehensive action of above-mentioned factors[13]. Studies that focus on the antibacterial activity of Ag-NPs when considering various nano sizes are limited[14].

The objective of this study was to obtain a better understanding of the antibacterial mechanism of action of Ag-NPs as a function of particle size[15]. Therefore, in this study, Ag-NPs of different sizes were synthesized using eco-friendly reagents, and their antibacterial properties were examined against *Vibrio natriegens* (*V*. *natriegens*) by determining the minimum inhibitory concentration (MIC), bacterial membrane damage, reactive oxygen species (ROS), and DNA damage. As one of the most abundant bacteria in the oceans, *V*. *natriegens* is easily attached to steel structures, such as hull and structural parts used for oil drilling and production. In our preliminary study, we showed that *V*. *natriegens* is a dominant bacterial species, however information on *V*. *natriegens* is limited. Therefore, in our study, we used *V*. *natriegens* as a model to promote the comparison of antibacterial activities of Ag-NPs with different particle sizes and different concentrations[16].

## Experimental procedures

### 2.1 Synthesis of Ag-NPs of different sizes

Ag-NPs of different sizes were synthesized using a green synthetic method and casein hydrolysate as a reducing reagent and sodium hydroxide (NaOH) as a catalyst. The preparation procedure was as follows: a batch of fresh sterile water (90 mL) containing casein hydrolysate (0.045 g) was prepared, followed by the addition of NaOH and aqueous silver nitrate (AgNO_3_) solution (10 mL), successively. The NaOH content and AgNO_3_ concentration ranged from 0.01 g to 0.06 g and 2 mM to 15 mM, respectively. The solutions were heated for 3 h at 60°C under mild stirring (90rpm) using a magnetic stirrer. Powdery substances were obtained after centrifugation, washing, and drying in a vacuum drying oven for 12 h at 60°C.

### 2.2 Characterization of Ag-NPs

X-ray diffraction measurements of Ag-NPs were performed using an X-ray diffractometer (XRD, the Netherlands, PANalytical B.V., X'Pert PRO) equipped with a Cu Kα X-ray source and operated at 40 kV and 40 mA. The optical properties and the dispersion stability of Ag-NPs were measured by UV-Vis absorption spectroscopy (Perkin Elmer, Lambda 35, Waltham, MA, USA) using a range of 300–600 nm. The particle size and nanostructure were characterized by transmission electron microscopy (TEM, JEOL, JEM 2100F, Tokyo, Japan) that was operated at 200 KeV.

After dilution of the nano-silver particles with deionized water, the particle size distribution of the nano silver particles was measured by a laser particle size analyzer (NanoBrook, 90plus Zeta, Holtsville, NY, USA).

### 2.3 Antibacterial activity of Ag-NPs

To investigate the bactericidal effect of Ag-NPs of different sizes, a single colony of *V*. *natriegens* was inoculated in 2216E medium (without antibiotics). The antibacterial activity of Ag-NPs was detected by MIC determinations. In brief, test tubes containing 2216E medium (10 mL) were inoculated with overnight bacterial cultures, then Ag-NPs solutions with dimensions of 10±5 nm, 30±5 nm, 60±5 nm, 90±5 nm and the concentrations ranging from 0.5 μg/ml to 15 μg/ml were added to each tube. The tubes were incubated overnight under shaking (120rpm) at 37°C. To assess bacterial growth, the increase in optical density at 600 nm (UV-Vis spectrophotometer) was measured for each tube. To further verify the antibacterial activity, the NP-treated bacterial culture was inoculated on Muller-Hinton agar (MHA) plates and sterilized at 37°C for 24 hours, and the minimum bactericidal concentration (MBC) was measured. The MBC is defined as the lowest concentration of NP that does not noticeable bacteria grow on agar plates [17].

ROS detection was carried out according to the method presented by Domínguez et al. In brief, 0.1 mL of bacterial suspension (OD600 = 1.0) in Hanks balanced salt solution (HBSS) was incubated with 0.5 mL nitroblue tetrazolium (NBT, 1 mg/mL) in the presence of AgNO_3_ solution or Ag-NPs which had been Illuminated for 30 min at 37°C in dark conditions. Then, 0.1 mL HCl (0.1M) was added to stop the reaction, and tubes were centrifuged at 1500 ×g indoor temperature for 10 min. The pellets were treated with dimethylsulphoxide (DMSO) to extract reduced NBT. Finally, 0.8 mL of HBSS was added and the OD was measured at 575 nm to assess intracellular ROS.

The effect of Ag-NP-induced damage to DNA molecules was evaluated by single cell gel electrophoresis using an Oxiselect comet assay kit (Cell Biolabs Inc., San Diego, CA, USA). Bacterial cells before and after Ag-NP treatment (25 ppm for 4 h) were collected by centrifuging at 700 X g for 2 min at 4°C. Cells were combined with comet agarose at 1:10 ratio (v/v), and spread over a glass slide that was transferred to 4°C in the dark for 15 min. Subsequently, the slides were carefully transferred to 25 mL of RIPA lysis buffer for 30–60 min at 4°C in the dark followed by induction using an alkaline solution for 30 min at 4°C and washing with pre-chilled Tris-boric acid (TBE) electrophoresis buffer. Glass slides were rinsed with cold 70% ethanol for 5 min, and air dried. Finally, Vista Green DNA dye was added to the bacterial sample and samples were observed using an inversed fluorescent microscope (Nikon Ecltpse Ti-E, Beijing, China).

When evaluating the surface morphology of the bacteria by TEM, bacteria were instilled on the carbon membrane support of 300 mesh at the early stage, then fixed with glutaraldehyde solution for 15 minutes, and finally dehydrated with 50%, 70%, 80%, 90% ethyl alcohol, and anhydrous ethanol. To prevent bacteria from being washed away during fixation and dehydration, the carbon membrane support was placed on the absorbent paper, and glutaraldehyde and alcohol were continuously soaked in the absorbent paper.

## Results and discussion

### 3.1 Particle characterization

**Fig 1:**
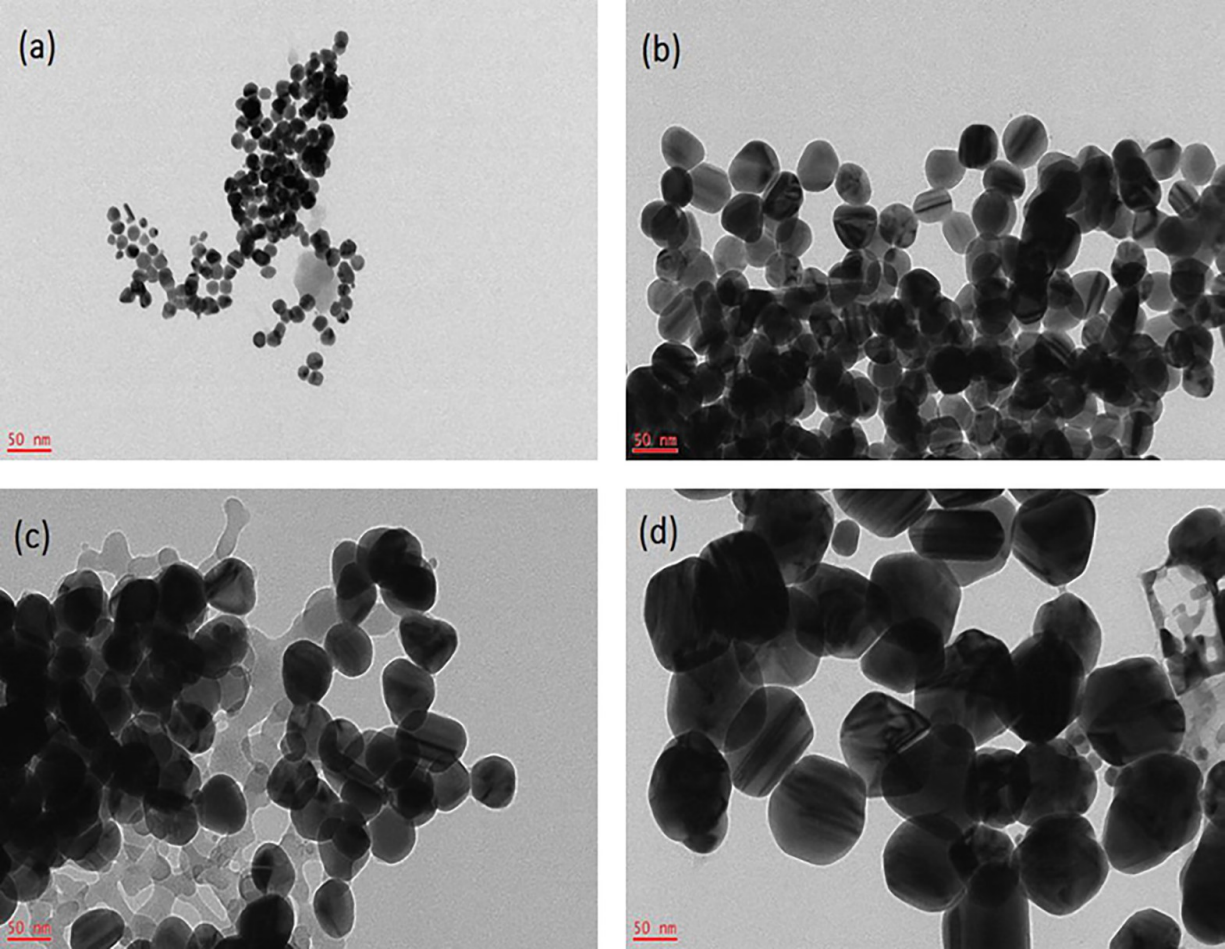
TEM photographs of different particle sizes of Ag-NPs. 10±5nm (a), 30±5nm (b), 60±5nm (c) and 90±5nm (d).

**Fig 2:**
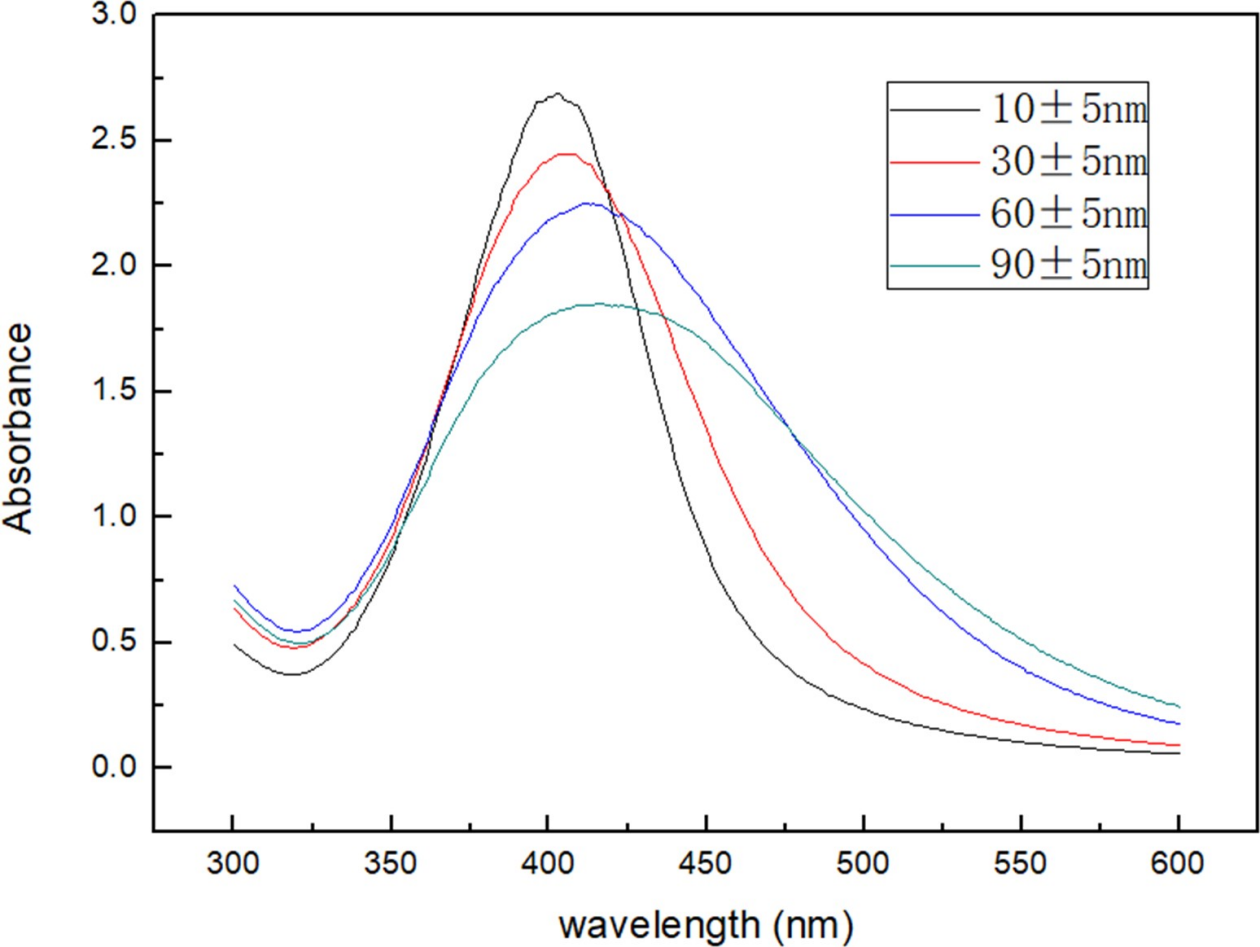
UV-Vis spectra with surface plasmon resonance showing the effect of AgNO_3_ (2-15mM) and NaOH (0.01–0.06g).

**Fig 3:**
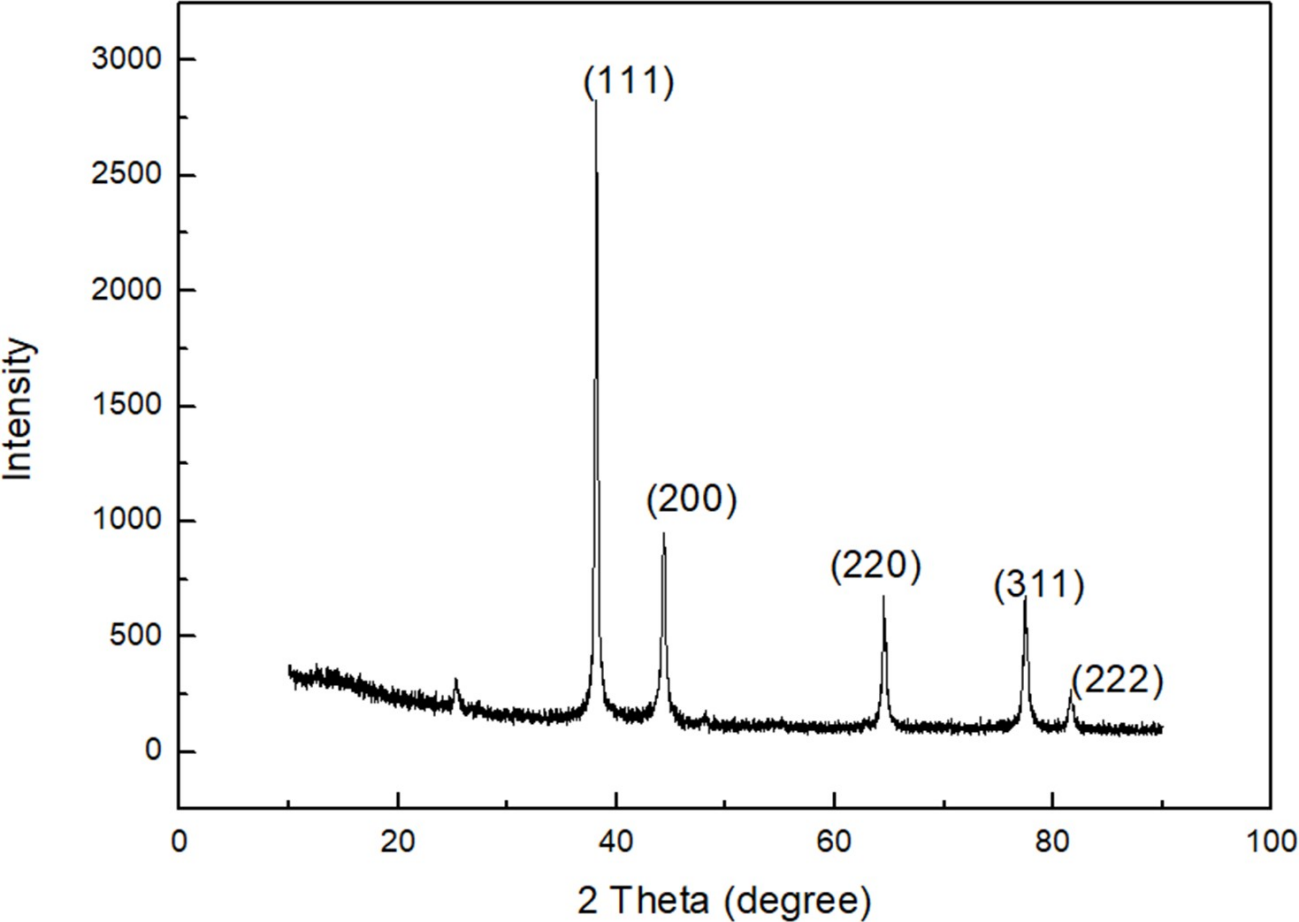
XRD spectrum of purified Ag-NPs.

By controlling the pH value of the reaction system, and the concentration of AgNO_3_, Ag-NPs of different particle sizes can be obtained (Fig 1). In this study, Ag-NPs of four different particle sizes were established and determined by TEM. In addition, by manually measuring the size of the particles, it was determined that the average sizes of the nanoparticles were 10 nm, 30 nm, 60 nm, 90 nm, and that the error range was about 5 nm. Fig 1 shows that Ag-NPs were spherical in shape, particles are uniform in size, and regular in shape. Particle sizes were 10±5 nm (Fig 1A), 30±5 nm (Fig 1B), 60±5 nm (Fig 1C), and 90±5 nm (Fig 1D), respectively. Ag-NPs were detected by UV-Vis spectrophotometry. The relationship between the position of the absorption peak and particle size is shown in Fig 2. Upon particle increase, the position of the absorption peak shifted to the long wave, and the larger the particle size, the more obvious the red shift phenomenon. As shown in Fig 3, the red shift of the absorption peak was the most significant when the particle size of Ag-NPs reached the maximum size of 90±5nm. Prepared Ag-NPs were represented by an XRD pattern (Fig 3). Fig 3 demonstrates that there are five diffraction peaks between 2θ = 20°-80° at Ag-NPs of 10nm, which was consistent with peaks of other particle sizes, and were applicable to all particle sizes. When compared to the standard pattern, they corresponded to the diffraction peaks of five crystal faces of the face-centered cubic metal silver. The corresponding crystal face indices from the inside to the outside were (111), (200), (220), (311), and (222). The spectrum showed no other impurity peaks, thereby illustrating that the relative structure of the prepared sample involved single-phase Ag-NPs with a cubical structure.

### 3.2 Distribution and dispersion stability of nano-silver in water

**Fig 4:**
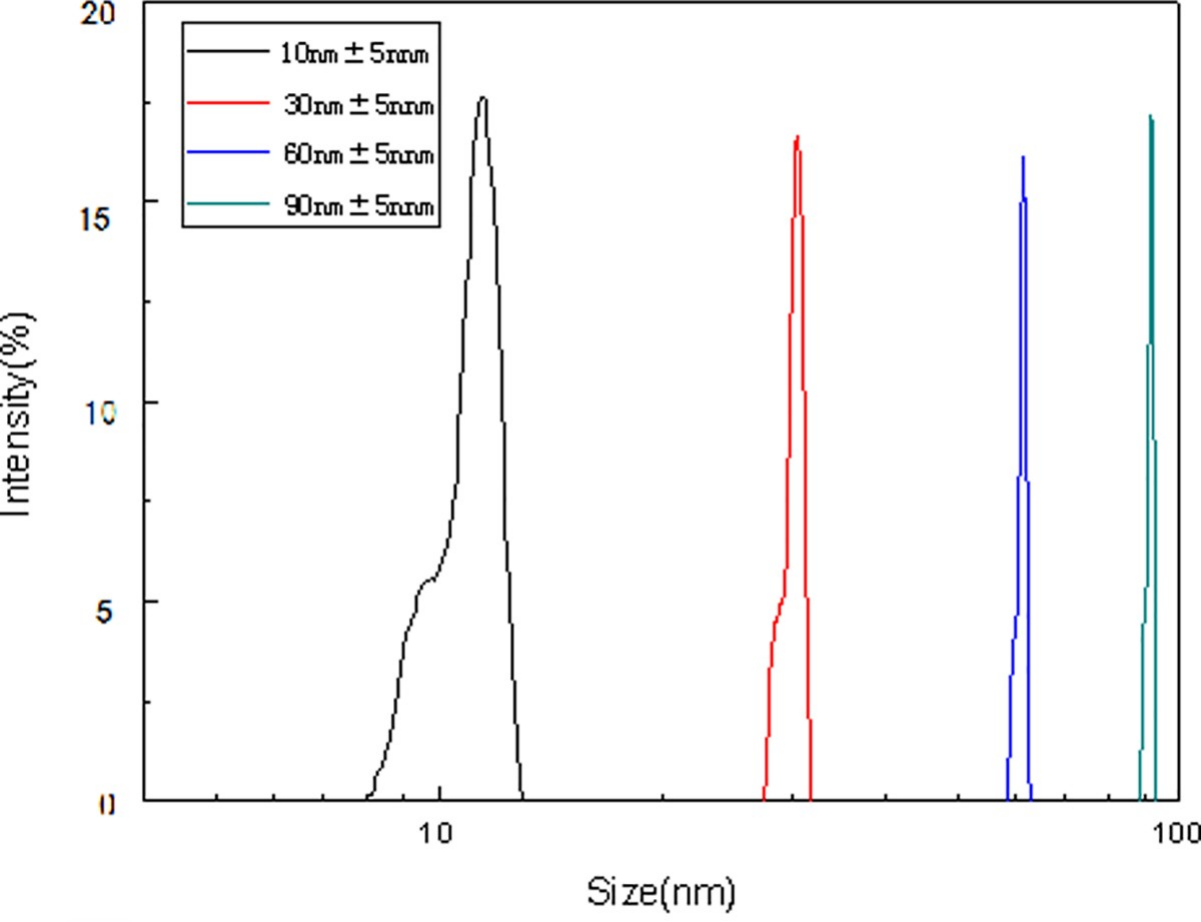
Particle size distribution of nano-silver in water.

**Fig 5:**
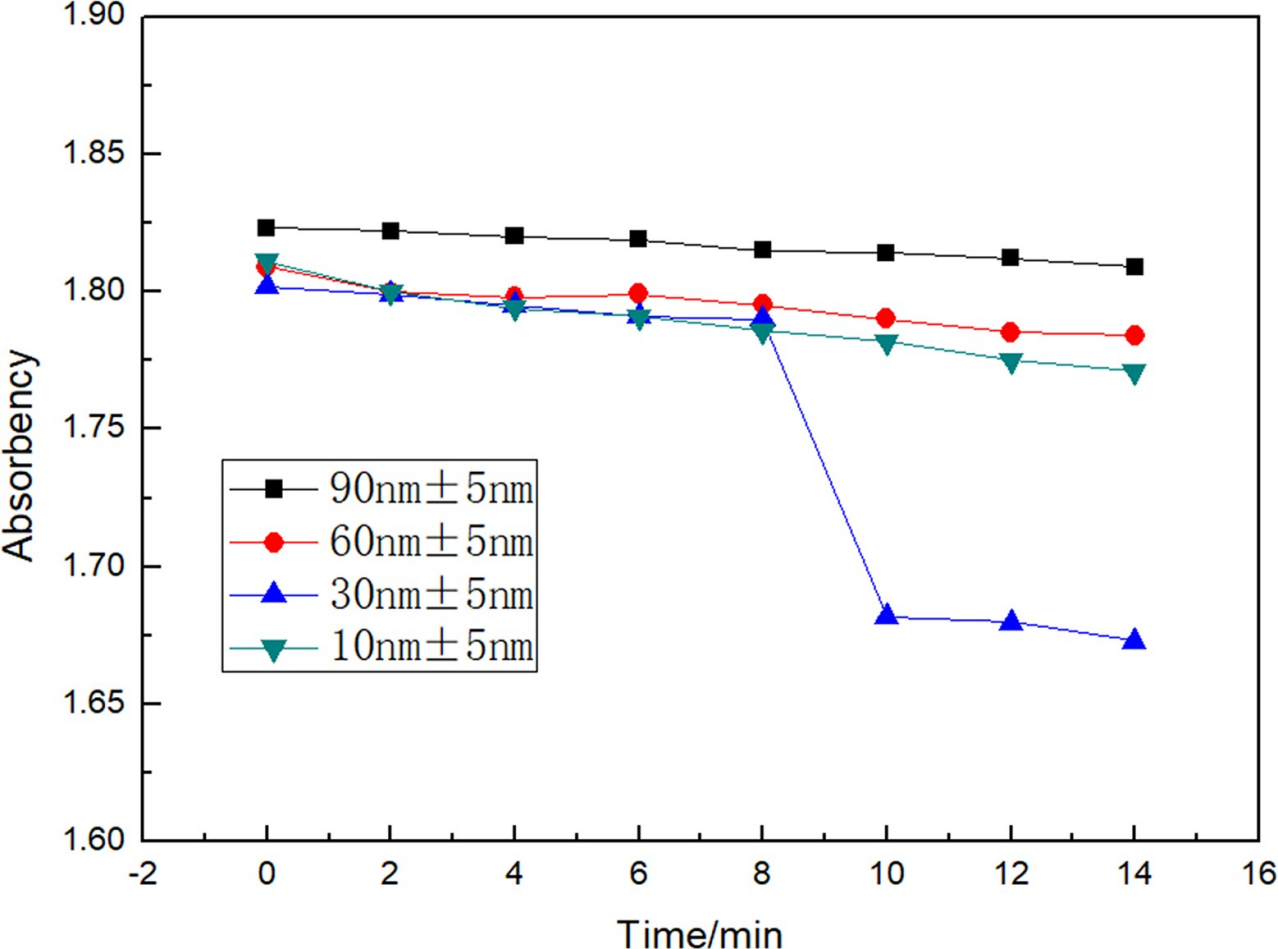
Dispersion stability diagram of nano-silver.

Fig 4 shows the particle size of nano-silver distribution in water. The figure shows that the particle size distribution of the nano-silver particles prepared were 10 nm, 30 nm, 60 nm, and 90 nm, respectively, and the particle size distribution is relatively uniform. The variation of the dispersion stability of nano-silver in a neutral aqueous medium over time is shown in Fig 5. Due to the influence of the particle size, shape, and surface properties, nano-silver particles are prone to agglomeration and deposition in water, therefore, the dispersion stability is linear with the time. Our findings showed that the smaller the size of the nanoparticles, the more obvious the phenomenon of agglomeration. This may be because the smaller the size of the nanoparticles, the larger the specific surface area, the higher the surface energy, the greater the intermolecular force, and therefore the poorer their dispersion stability. The experimental medium is neutral water, therefore, the main reason for the dispersion stability of the aqueous phase system may be the migration of nano-silver particles, and the changes in particle size may have little effect. In addition, the dispersion stability of nano-silver at these sizes may show small differences.

### 3.3 Minimum inhibitory concentration and minimum bactericidal concentration

Ag-NPs can effectively inhibit the growth of *V*. *natriegens*. In brief, the antibacterial activity of Ag-NPs of different sizes was obtained by determining MIC and MBC as shown in Table 1. The error range of Ag-NPs particle sizes was about ±5 nm (Table 1). We demonstrated that 10±5 nm Ag-NPs can completely inhibit bacteria at a lower MIC (1.0 μg/ml), whereas a particle size of about 90±5 nm can inhibit bacteria at a higher concentration (11.5 μg/ml). Among the different particle sizes, we tested the observed sensitivity toward Ag-NPs and found that 10±5 nm > 30±5 nm > 60±5 nm > 90±5 nm. We also determined the MBC of all particle sizes, the MBC for 10±5 nm Ag-NPs was the lowest (1.1 μg/ml) and that of 90±5 nm Ag-NPs was the highest (11.7 μg/ml). The results of MBC and MIC were consistent, thereby indicating that the antibacterial properties of Ag-NPs were affected by size. In this study, we found that the smaller the particle size, the better the bactericidal effect.

**Table 1:** Minimum inhibitory concentration and minimum bactericidal concentration for different particle sizes.

Particle size	MIC (μg/ml)	MBC (μg/ml)
10±5nm	1.0	1.1
30±5nm	2.4	2.6
60±5nm	7.2	7.2
90±5nm	11.5	11.7

Abbreviations: MIC, minimum inhibitory concentration; MBC, minimum bactericidal concentration.

## 3.4 Reactive oxygen species and DNA

**Fig 6:**
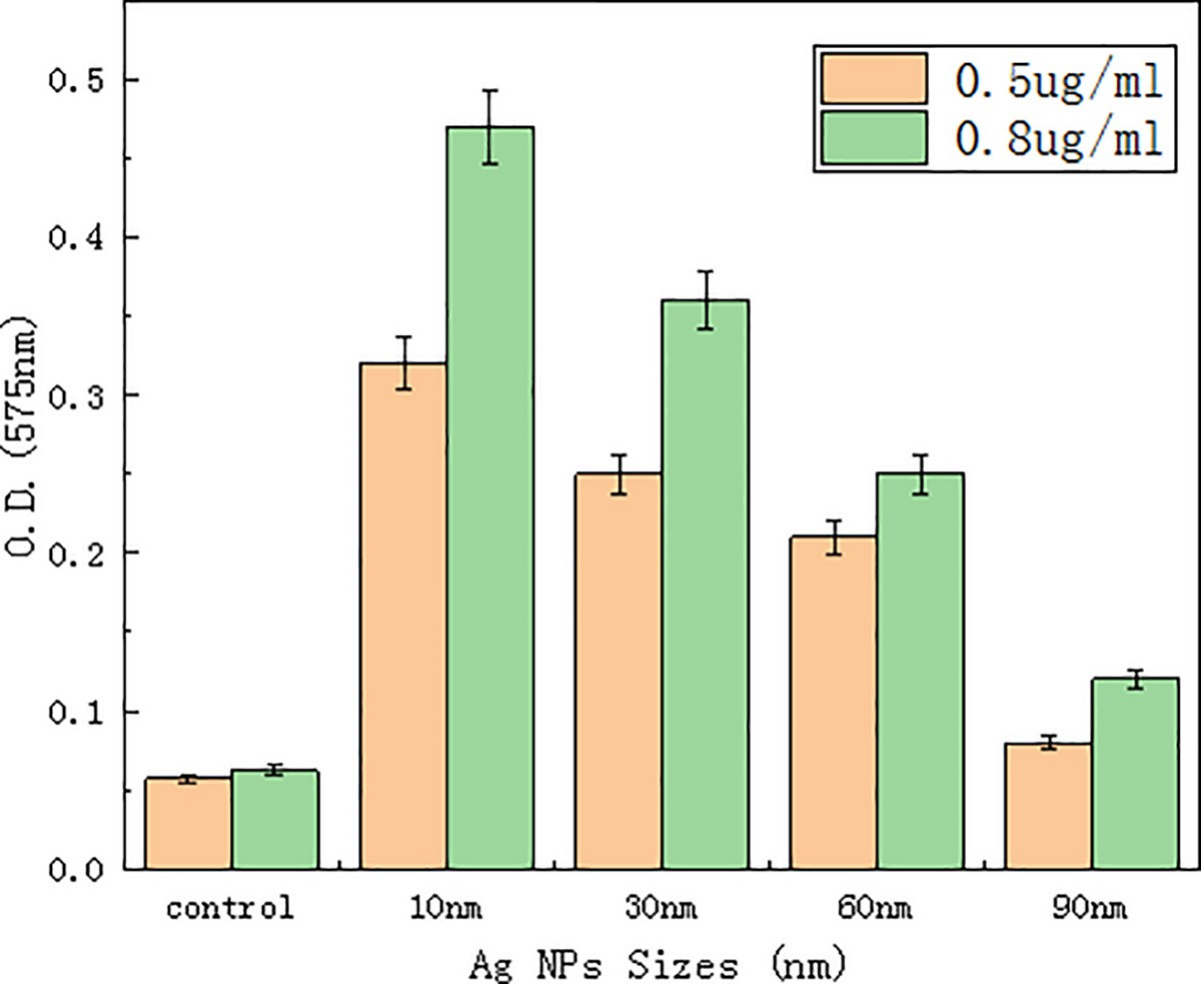
Reactive oxygen species in *V*. *natriegens* after treatment with various concentrations and particle sizes of Ag-NPs in the presence or absence of nitroblue tetrazolium.

**Fig 7:**
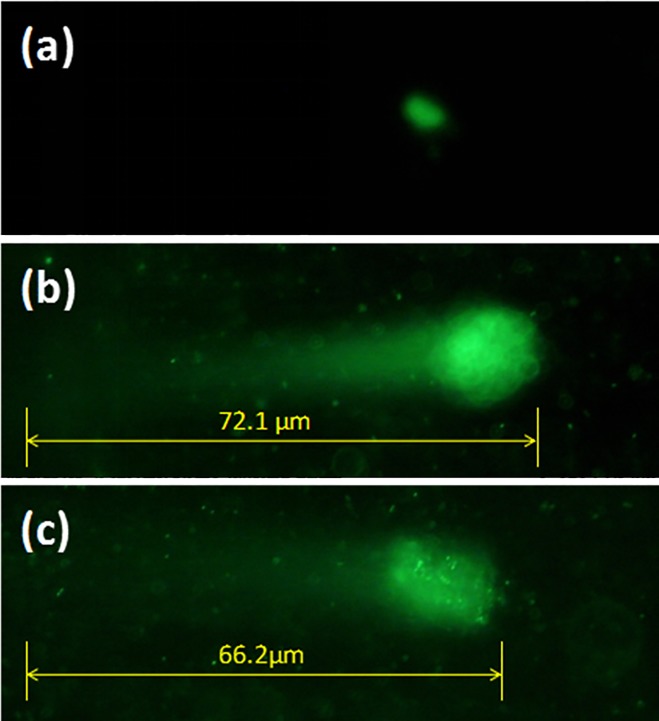
**Comet assay images of bacteria after different treatments:** control (a); concentration of Ag-NPs at 0.5 μg/ml (b); concentration of Ag-NPs at 0.8 μg/ml (c).

The production of ROS was assessed in bacterial strain (*V*. *natriegens*) by treating *V*. *natriegens* with different concentrations and different sizes of Ag-NPs. When aerobic bacteria during metabolism suffer from various harmful stimuli, excessive ROS [18] are produced, including superoxide radical O_2_^-^[19, 20]. The degree of oxidation exceeds the ability of various active enzymes and antioxidants in bacteria to scavenge ROS, which leads to an imbalance between oxidation and antioxidant systems, thereby causing bacterial damage[21]. To determine the extent of ROS-related damage, equal amounts of NBT were added to different sizes and concentrations of Ag-NPs. Fig 6 shows that except for the control, at similar concentrations, increasing Ag-NP particle size results in decreasing O.D._575nm_ values. Because the NBT content was constant, we concluded that when the Ag-NP size decreased, more NBT was gradually redox-reduced, that is, more ROS was produced by bacteria. When Ag-NP sizes were similar, O.D._575nm_ values increased when the concentration of Ag-NPs increased. During oxidative stress responses, various macromolecular substances, such as DNA, proteins, etc., that make up cellular tissues may undergo various degrees of oxidation. To verify ROS-induced damage to bacterial DNA[22], the comet assay approach was employed[23, 24]. The comet assay is a sensitive approach that has been developed in recent years to quantify DNA damage at the single cell level. After continuous improvement, the genetic damage detected by this method has become a sensitive marker for identifying genotoxic substances, and plays an important role in the carcinogenic mechanism of action and environmental pollution monitoring and evaluation. To discriminate injury of cellular genetic material by detecting damaged DNA double-strands, it was found that the more severe the damage to DNA, the more chains and fragments were produced, and the longer the distance the DNA migrated under similar electrophoresis conditions. Data obtained from the comet assay by treatment with 10±5 nm Ag-NPs at concentrations of 0.5 μg/ml and 0.8 μg/ml are shown in Fig 7. Compared to the control, the length of the “comet” was significantly longer at the concentration of 0.5 μg/ml compared to an Ag-NPs concentration of 0.8 μg/ml. Together, these findings indicated that bacterial DNA damage was more severe at a concentration of 0.5 μg/ml compared to a concentration of 0.8 μg/ml. Thus, it seemed that ROS produced by bacteria can cause DNA damage, and the higher the concentration of ROS produced, the more severe the DNA damage.

### 3.5 Bacterial morphology

**Fig 8:**
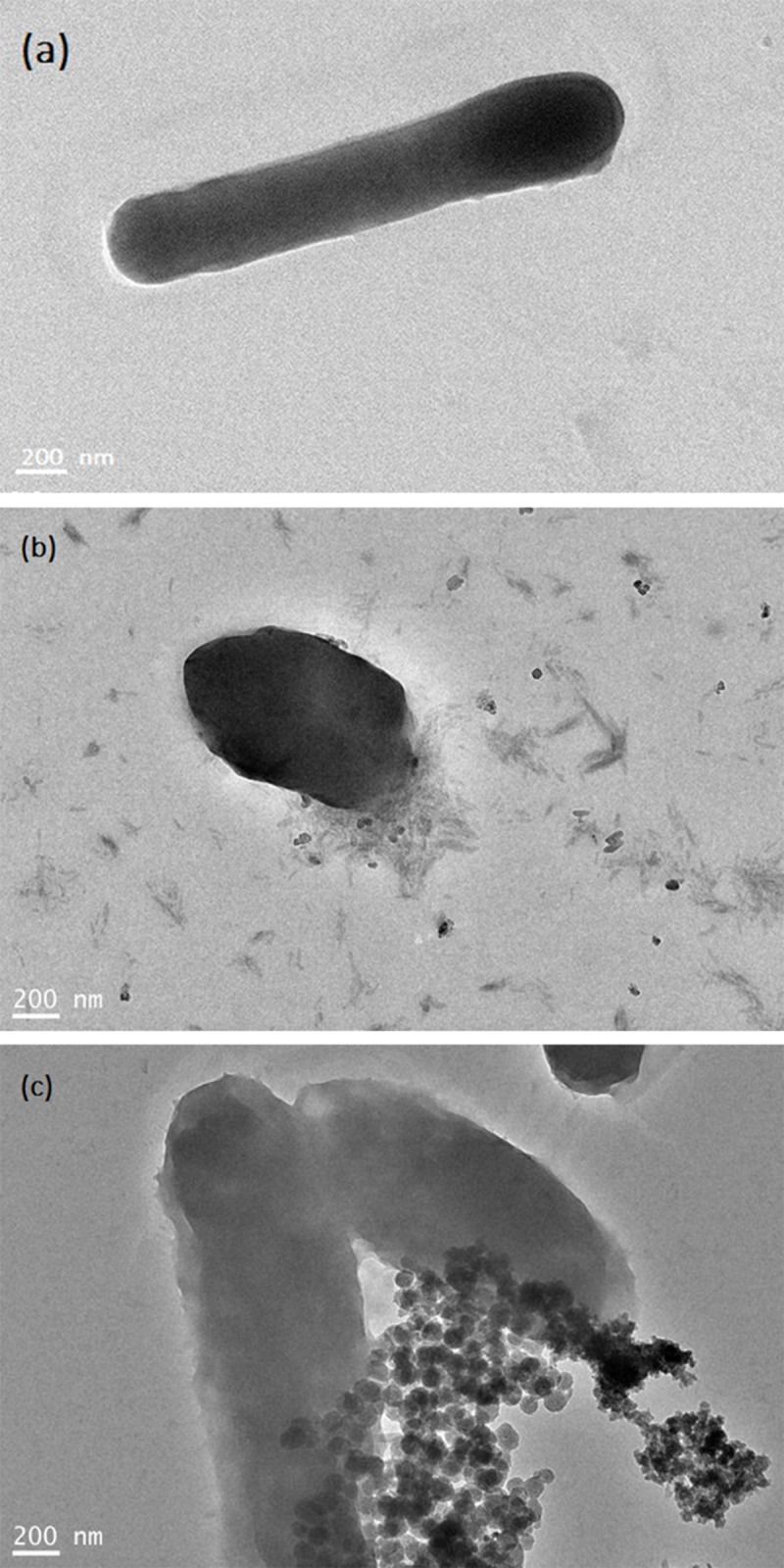
**TEM images of** a control (a), membrane rupture (b), and Ag-NPs penetrating into cells (c).

The inhibitory effect of Ag-NPs on bacteria is also reflected in the destruction of cell integrity[13]. Fig 8 represents a TEM image of a bacterium before and after the treatment with Ag-NPs. When compared with the untreated cell structure (Fig 8), we found that Ag-NPs can enter the cellular body, in one mode causing cell membrane[10,25] rupture and in other mode directly infiltrating into the cell to damage it. *V*. *natriegens* is a Gram-negative bacterium, about 4 μm in length[14]. It represents a type of active aerobic bacteria that when compared to Gram-positive bacteria has no spores and no capsules[12,26]. The bacteria are nitrogen-fixing bacteria that are commonly found in marine and estuarine environments. *V*. *natriegens* has a fast propagation rate, and the formation of a biofilm on the surface of the material is convenient, such as the rolled steel aluminum product.

## Conclusions

Our results strongly support the findings that Ag-NPs are potential antibacterial agents. Ag-NPs of different particle sizes were synthesized by controlling the concentration of AgNO_3_ and NaOH. In this study, we used *V*. *natriegens* as a model to promote the comparison of antibacterial activities of Ag-NPs with different particle sizes and different concentrations. The data indicated that MIC and MBC values were the lowest when Ag-NPs were roughly 10±5 nm in size, i.e., the particle size was the smallest, 1.0 μg/ml and 1.1 μg/ml, respectively. Furthermore, the determination of bacterial ROS as well as comet assays have shown that the smaller the particle size of Ag-NPs, the more bacterial cell damage, and the more obvious the oxidative stress response (ROS). ROS caused DNA damage, and cell membrane rupture is the main mechanism of antibacterial activity of Ag-NPs. Thus, our findings may have significant implication in antibacterial therapy.
